# Supplementary material for: Female genital lichen sclerosus is connected with a higher depression rate, decreased sexual quality of life and diminished work productivity
Source: PLoS One. 2023 Apr 25;18(4):e0284948. doi: 10.1371/journal.pone.0284948 (PMC10128971; doi:10.1371/journal.pone.0284948)
Supplement: Attachment — Submitted filename: Comments PLoS 1.doc [file pone.0284948.s001.doc]

This paper reports a case-control study on lichen sclerosus and assesses a domain of the disease not in deep studied so far. Additionally, conclusion are in agreement with my own opinion on the disease. Moreover, it is easy to read and understand, reaching the goal of clarity I like in assessing articles.

I have some issues to be pointed out:

1. Criteria for diagnosing LS.

2. Specifying if some treatment for LS have been administered or not at the time of respondents’ answers, as it might affect them.

3. Describe best cases and controls characteristics. I understand you have matched controls, but you have not reported any planned design for doing it. Ideally, time frame in which case and controls have been enrolled would be the same and both cases and controls would came from same population. Finally, case-control studies are usually retrospective. Is this a retrospective study?

4. A structured abstract would be able to transfer the information of a case control study.

I thank you in advance.

Your,

UI
