# Supplementary material for: Female genital lichen sclerosus is connected with a higher depression rate, decreased sexual quality of life and diminished work productivity
Source: PLoS One. 2023 Apr 25;18(4):e0284948. doi: 10.1371/journal.pone.0284948 (PMC10128971; doi:10.1371/journal.pone.0284948)
Supplement: Attachment — Submitted filename: Response to Reviewers.docx [file pone.0284948.s002.docx]

Dear Dr. Alessandro Favilli,

My co-authors and I were pleased to receive your response of 27 February 2023 providing helpful feedback and inviting us to resubmit our paper after minor revisions. We are very grateful for your time and effort involved in reviewing our manuscript.

Enclosed you will find our revised paper ‘Female genital lichen sclerosus is connected with a higher depression rate, decreased sexual quality of life and diminished work productivity.’ for your consideration. We sought to improve the content and presentation of the manuscript following the insightful feedback we received. The actions taken in response to individual comments are explained further below.

Journal Requirements:

1. “Please ensure that your manuscript meets PLOS ONE's style requirements, including those for file naming. The PLOS ONE style templates can be found at
   <https://journals.plos.org/plosone/s/file?id=wjVg/PLOSOne_formatting_sample_main_body.pdf> and 
   <https://journals.plos.org/plosone/s/file?id=ba62/PLOSOne_formatting_sample_title_authors_affiliations.pdf>”

We reviewed the PLOS ONE's style requirements, changed the file naming and saved figure files as “Fig1.tif”, “Fig2.tif”.

2. “Please ensure that you have specified (1) whether consent was informed and (2) what type you obtained (for instance, written or verbal, and if verbal, how it was documented and witnessed). If your study included minors, state whether you obtained consent from parents or guardians. If the need for consent was waived by the ethics committee, please include this information.”

We specified that written informed consent was obtained from all participants of the study. We did not include minors in our study. We elaborate on the ethics committee’s opinion under point 4 below.

3. “We note that you have stated that you will provide repository information for your data at acceptance. Should your manuscript be accepted for publication, we will hold it until you provide the relevant accession numbers or DOIs necessary to access your data. If you wish to make changes to your Data Availability statement, please describe these changes in your cover letter and we will update your Data Availability statement to reflect the information you provide.”

We have stored our data in the open access repository Zenodo. The DOI to access our data is 10.5281/zenodo.7782999

4. “Please include your full ethics statement in the ‘Methods’ section of your manuscript file. In your statement, please include the full name of the IRB or ethics committee who approved or waived your study, as well as whether or not you obtained informed written or verbal consent. If consent was waived for your study, please include this information in your statement as well.”

Information about the ethics committee’s opinion was included in the ‘Methods’ section. The study was exempt from research ethics review based on the formal opinion of the Bioethics Committee of the Medical University of Lodz of 13 December 2022 (No. RNN/282/22/KE). We enclose the original document along with its translation.

5. “Please review your reference list to ensure that it is complete and correct. If you have cited papers that have been retracted, please include the rationale for doing so in the manuscript text, or remove these references and replace them with relevant current references. Any changes to the reference list should be mentioned in the rebuttal letter that accompanies your revised manuscript. If you need to cite a retracted article, indicate the article’s retracted status in the References list and also include a citation and full reference for the retraction notice.”

We reviewed the reference list following PLOS ONE requirements.

6. We also reassessed the status of the women who declared in the Work Productivity and Activity Impairment: General Health (WPAI:GH) questionnaire not to be working. Seven of the participants were not professionally active. There were 3 pensioners, 2 students and 2 women who did not work due to unknown reasons. We revised it in the manuscript.

Reviewer #2:

1. “Criteria for diagnosing LS.”

We explained in the revised version of the paper that the study group included individuals in whom the diagnosis of LS was set clinically by the doctor, based on characteristic features of skin lesions and concomitant symptoms. In a few cases the diagnosis was additionally confirmed by a skin biopsy.

2. “Specifying if some treatment for LS have been administered or not at the time of respondents’ answers, as it might affect them.”

We specified that 30 women in our study group declared to be using topical corticosteroids, 4 of whom only milder potency ones, 6 applied calcineurin inhibitors, 16 mentioned using other treatment options (e.g. photodynamic therapy, laser, platelet rich plasma) and 3 did not use any form of treatment.

3. “Describe best cases and controls characteristics. I understand you have matched controls, but you have not reported any planned design for doing it. Ideally, time frame in which case and controls have been enrolled would be the same and both cases and controls would came from same population. Finally, case-control studies are usually retrospective. Is this a retrospective study?”

The study is a retrospective case-control study. The process of enrollment to the study and control group took place between January and December 2021. Cases and controls were matched based on gender, age, occupation and place of residence. The characteristics of the study and control group are presented graphically on the chart (Fig 1).

4. “A structured abstract would be able to transfer the information of a case control study.”

We added information about the nature of the study – retrospective case-control study – in the abstract of the paper.

We look forward to your feedback on the revised version of the manuscript.

Kind regards,

Olga Jabłonowska
